# Evaluating the impact of a polypharmacy Action Learning Sets tool on healthcare practitioners’ confidence, perceptions and experiences of stopping inappropriate medicines

**DOI:** 10.1186/s12909-022-03556-8

**Published:** 2022-06-27

**Authors:** Cindy Faith Brooks, Anastasios Argyropoulos, Catherine Brigitte Matheson-Monnet, David Kryl

**Affiliations:** 1 NIHR ARC Wessex and School of Health Sciences, University of Southampton, Southampton, SO17 1BJ UK; 2 Public Health, Swindon Borough Council, Civic Offices, Swindon, SN1 2JH Wiltshire UK; 3 School of Health Sciences, University of Southampton, Southampton, SO17 1BJ UK

**Keywords:** Action learning sets tool, Polypharmacy, Education, Healthcare practitioners, GP, Pharmacists, Pharmacy professionals

## Abstract

**Background:**

Issues of medication adherence, multimorbidity, increased hospitalisation risk and negative impact upon quality of life have led to the management of polypharmacy becoming a national priority. Clinical guidelines advise a patient-centred approach, involving shared decision-making and multidisciplinary team working. However, there have been limited educational initiatives to improve healthcare practitioners’ management of polypharmacy and stopping inappropriate medicines. This study aimed to evaluate the impact of a polypharmacy Action Learning Sets (ALS) tool across five areas: i. healthcare practitioners’ confidence and perceptions of stopping medicines; ii. knowledge and information sources around stopping medicines; iii. perception of patients and stopping medicines; iv. perception of colleagues and stopping medicines and v. perception of the role of institutional factors in stopping medicines.

**Methods:**

The ALS tool was delivered to a multi-disciplinary group of healthcare practitioners: GPs [*n* = 24] and pharmacy professionals [*n* = 9]. A pre-post survey with 28 closed statements across five domains relating to the study aims [*n* = 32] and a post evaluation feedback survey with 4 open-ended questions [*n* = 33] were completed. Paired pre-post ALS responses [*n* = 32] were analysed using the Wilcoxon signed-rank test. Qualitative responses were analysed using a simplified version of the constant comparative method.

**Results:**

The ALS tool showed significant improvement in 14 of 28 statements in the pre-post survey across the five domains. Qualitative themes (QT) from the post evaluation feedback survey include: i. awareness and management of polypharmacy; ii. opportunity to share experiences; iii. usefulness of ALS as a learning tool and iv. equipping with tools and information. Synthesised themes (ST) from analysis of pre-post survey data and post evaluation feedback survey data include: i. awareness, confidence and management of inappropriate polypharmacy, ii. equipping with knowledge, information, tools and resources and iii. decision-making and discussion about stopping medicines with colleagues in different settings.

**Conclusions:**

This evaluation contributes to developing understanding of the role of educational initiatives in improving inappropriate polypharmacy, demonstrating the effectiveness of the ALS tool in improving healthcare practitioners’ awareness, confidence and perceptions in stopping inappropriate medicines. Further evaluation is required to examine impact of the ALS tool in different localities as well as longer-term impact.

## Background

Polypharmacy refers to the routine use of at least five or more medicines [1–3]. It is linked with multimorbidity and is more prevalent among older people [1, 4, 5]. Associated issues include medication adherence, increased risk of unplanned hospitalisation, greater risks of adverse drug reactions and negative impact on quality of life [6–9].

To address issues relating to polypharmacy, a distinction can be made between ‘appropriate’ and ‘problematic’ polypharmacy. Appropriate polypharmacy refers to the optimisation of medicine using high quality-evidence to prescribe for a person with complex and/or multiple conditions, which is contributing to increased life expectancy and better quality of life. Problematic polypharmacy refers to inappropriate prescribing of multiple medications and is associated with greater risk of drug interactions and problematic reactions [8, 10].

A review of barriers and enablers to stopping inappropriate medicines by the University of York et al. [5], identified various contributory factors for patients and practitioners. For patients, factors include emotions (e.g. perceptions of illness), purpose and goals for stopping and social factors (e.g. influence of health professionals or family) [11, 12]. For practitioners, factors include confidence, perceptions of consequences and risk, role of environmental factors (e.g. time and resources) and social factors (e.g. patient willingness/unwillingness) [13, 14].

Clinical guidelines by NICE [9, 15], the General Medical Council [16] and The Royal Pharmaceutical Society [17] advocate a patient-centred approach, involving shared decision-making to manage inappropriate polypharmacy. More specifically, emphasising the importance of informed patient consent in the process of stopping inappropriate medicines, involving both the individual (level of patient-prescriber) and the context (i.e. wider system level of care) [13, 14].

Other recommendations include the adoption of a multidisciplinary team approach, organised medication reviews, and the use of a screening tool (e.g. STOPP/START) and individually tailored management plans for patients, to accommodate their aspirations, preferences and needs [5, 15]. However, the extent to which practitioners can involve patients in shared decision making, for example in medication reviews, is often impacted by contextual environmental factors, such as time pressures and workload commitments, requiring various levels of time and involvement and dependent upon patient need and complexity [18].

Issues of medication adherence, multimorbidity, increased hospitalisation risk and negative impact upon quality of life have led to the management of polypharmacy becoming a national priority. However, there have been a limited number of educational initiatives to improve healthcare practitioners’ management of inappropriate polypharmacy and in stopping inappropriate medicines.

### Interventions to address inappropriate polypharmacy

A range of interventions have been advanced to address the issues associated with inappropriate polypharmacy [19, 20]. These can be distinguished as either interventions to improve appropriate polypharmacy or interventions to aid deprescribing [5]. Improving appropriate polypharmacy focuses upon the optimisation of medicine use for multiple or complex conditions, by prescribing medicine in relation to the best evidence [8, 21]. Deprescribing describes the process required to aid the safe reduction of inappropriate medicines [21].

Interventions to improve appropriate polypharmacy include professional (e.g. using health technology or educational approaches), organisational (e.g. medication reviews by pharmacists, physicians or nurses) or multidimensional pharmaceutical focused approaches [5, 19, 20].

More specifically, as a subset of interventions to improve polypharmacy, interventions to aid deprescribing, that is, the safe removal/stopping of medicines [22], can include screening tools or specifications (e.g. Beers, Medication Appropriateness Index, STOPP (*screening tool of older people’s prescriptions*), START (*screening tool to alert to right treatment*), more defined approaches to deprescribing (The 7 Steps), and/or the Drug burden index [1, 5, 13, 23].

Despite the range of interventions to improve polypharmacy and to aid deprescribing, there are limited examples of research into the contribution of educational interventions to improve healthcare practitioners’ perceptions and confidence in managing polypharmacy. Pitkälä et al. [24] investigated the impact of a learning intervention to identify potentially unsafe medications, for nurses responsible for comprehensive care of appropriate medication treatment among 227 residents in 20 wards of assisted living facilities compared to a control group of nurses not receiving the intervention. They concluded that the learning methods can decrease the use of unsafe medications, sustain quality of life and decrease hospitalisation for residents in supported living settings.

Bregnhøj et al. [25] undertook a combined intervention programme to reduce inappropriate prescribing in elderly patients in primary care with GPs randomised to either (1) a combined intervention consisting of an interactive educational meeting plus feedback on participating patients’ medication, (2) a single intervention with an interactive educational meeting or (3) a control group (no intervention). They found that the combined intervention can enhance the suitability of prescribing for elderly patients who are vulnerable to polypharmacy.

Mecca et al. [26] undertook a mixed methods evaluation of an interprofessional polypharmacy and deprescribing intervention designed to decrease the pharmaceutical risk in an older veterans’ polypharmacy clinic. The intervention aimed to evaluate residents’ knowledge of polypharmacy and perceptions of the intervention against an internal comparison group. The sample involved internal medicine residents and nurse practitioner residents as well as veterans aged 65, who were taking 10 medications. After a period of 6 months, they found that the intervention group had a substantially greater improvement in test scores in comparison to the control group. Qualitative findings from focus groups with residents, showed that residents perceived improvements in knowledge and skills. Interprofessional experience gained through the intervention was also acknowledged to be of benefit to participants. In addition, with respect to veterans, there was a median of 15 medications (Interquartile Range, ((IQR 12–19)) and a median of 2 medications (IQR 1–3) stopped. They concluded that the intervention had been an effective form of post-graduate training in polypharmacy management and deprescribing.

The Royal Pharmaceutical Society [17] raises the need for evidence-based tools to improve healthcare professionals’ confidence in stopping medicines safely. Moreover, it is recognised that further research and evaluation are required to understand the impact of such educational initiatives in improving inappropriate polypharmacy particularly in general practice and community pharmacy [8, 17]. This paper addresses the aforementioned areas and aims to improve understanding of the role of educational initiatives in addressing inappropriate polypharmacy by conducting an independent evaluation of the impact of a polypharmacy educational ALS tool upon healthcare practitioners’ confidence, perceptions and experiences of stopping inappropriate medicines.

## Methods

### Aims of the study

This paper reports on the findings of an independent evaluation of the ALS tool to ascertain its impact across five areas: i. healthcare practitioners’ confidence and perceptions of stopping medicines; ii. knowledge and information sources around stopping medicines; iii. perception of patients and stopping medicines; iv. perception of colleagues and stopping medicines and v. perception of the role of institutional factors in stopping medicines [27]. The evaluation also sought to understand respondents’ expectations, comprehension, what they found helpful/unnecessary, prospective application to practice and whether they would recommend it to colleagues.

### Setting

ALS sessions were lecture-based with interactive activities involving both small and whole group discussions as well as individual homework (i.e. work that participants were required to complete between sessions). In comparison to traditional ALS approaches, the ALS homework activities were not undertaken collectively as a group by participants, but individually within their own practice. Participants then had the opportunity to feedback and discuss their homework at the following session (Table 1).

**Table 1:** Structure of ALS sessions

Session	Focus
•	Introduced polypharmacy by using lecture and video format to depict issues associated with inappropriate polypharmacy. Group work involved discussing participants reflections of the challenges to stopping inappropriate medicines within their own practice. As homework, participants were encouraged to apply learning from the session to their practice before the next session
•	Focused upon participant experiences of undertaking a medication review and guidance on shared decision making as well as introducing different tools to assist in stopping inappropriate polypharmacy. Homework involved applying learning to a consultation with a patient
•	Involved a review of participants experiences of shared decision making and discussion with a Consultant Geriatrician about what went well and how this could be improved

### Data collection

The evaluation consisted of a pre-post polypharmacy ALS survey collecting quantitative data [*n* = 32] and a post evaluation feedback survey collecting both qualitative and quantitative data [*n* = 33].

### Pre and post ALS survey

The pre-post polypharmacy ALS survey was administered immediately before the first and immediately after the last ALS session. It measured participants’ confidence and perceptions in relation to 28 statements across five domains relating to the study aims: i. perceptions around stopping medicines; ii. knowledge and information sources iii. perception of patients and stopping medicines, iv. perception of colleagues and stopping medicines and v. institutional factors [27].

The statements were structured in a five-point Likert scale (1 = strongly disagree, 2 = disagree, 3 = neither agree or disagree, 4 = agree, 5 = strongly agree).

### Post evaluation feedback survey

The evaluation feedback survey consisted of 4 open-ended questions and 4 closed-ended questions. It was given to participants immediately after the last ALS session. It evaluated participants overall perceptions of the ALS, including their expectations, comprehension, what they found helpful/unnecessary, prospective application to practice and whether they would recommend it to colleagues.

### Sample

A total of 40 respondents completed a pre and/or post survey, from which a matched sample of 32 respondents completed both a pre and post survey. A sample of 33 respondents completed the post evaluation feedback survey, which included the 32 pre-post matched survey respondents. Respondents were requested to indicate their role, whether they were an independent prescriber (and if yes, the number of years as an independent prescriber). Of the 32 respondents; 24 identified as GPs, 6 as pharmacists, 1 as a pharmacy technician and 1 as a medicines’ management pharmacist. The majority (26/32), indicated that they were an independent prescriber (all the GPs and 2 pharmacy professionals); 6 reported they had been an independent prescriber for 1–5 years, 6 reported they had been an independent prescriber for 10–14 years, 5 for 30 years and over, 3 for 15–19 years, 2 for 20–24 years, 2 for 25–29 years, and 2 did not state the number of years.

### Data analysis

Data from the pre-post ALS survey and post ALS evaluation feedback survey were analysed independently, and then compared to show areas of conceptual synthesis in accordance to the areas outlined in the aims of the study.

### Analysis of pre-post ALS survey

For each of the 28 ALS survey statements, pre-ALS and post-ALS median and interquartile ranges were computed. Mean and standard deviation (SD) are also presented for the sake of completeness. Paired pre-ALS and post-ALS responses to the statements were compared and tested for change using the Wilcoxon signed-rank test. A non-parametric test was selected because data were of ordinal nature and not normally distributed. Statistical significance was determined by a probability of less than 0.05, so the probability *p* < 0.05 indicates that the difference is statistically significant. Data were analysed using SPSS 24.0. Effect size was computed for each statement in to assess the significance of the difference in participants’ pre-ALS and post-ALS responses to the statements. The Wilcoxon signed-rank test absolute value of the Z-score $\left|z\right|$ and the number of total observations included in the analysis of each questionnaire item $n$ were used in the effect size calculation $r$ [28, 29].

$$r=\frac{\left|z\right|}{\sqrt{n}}$$

The division of $\left|z\right|$ with a function of $n$ removes the effect of sample size from the effect size estimate and constitutes the effect size independent of sample size [30]. According to Cohen [28], effect sizes of $r=0.1$, $r=0.3$ and $r=0.5$ are respectively considered as small, medium and large.

### Analysis of post evaluation feedback survey

The qualitative responses were analysed using a simplified version of the constant comparative method [31]. Themes are described in turn and illustrated through verbatim representative extracts from the survey. The extracts are labelled according to the type of participant: pharmacy professional or GP.

Descriptive statistics and data analysis were conducted using MS Excel for quantitative data.

## Results

### Pre-post survey

The ALS tool demonstrated significant improvements in 14 statements relating to all five domains (D1-5). Pre and post descriptive statistics by question and/or domain are depicted in Tables 2, 3 and 4.

**Table 2:** Pre and post descriptive statistics by question for each domain

Domain	Questions	Pre			Post			Wilcoxon Signed Rank		
		mean (SD)	median (IQR)	mean (SD); median (IQR)	mean (SD)	median (IQR)	mean (SD); median (IQR)	Z score	*p* value*	effect size (r)
Perceptions around stopping medicines	Q1	3.63 (0.87)	4.00 (3.00–4.00)	4.16 (0.53); 4.25 (3.81–4.50)	4.09 (0.53)	4.00 (4.00–4.00)	4.44 (0.37); 4.25 (4.25–4.75)	-2.696	0.008	0.34
	Q2	4.66 (0.60)	5.00 (4.00–5.00)		4.88 (0.42)	5.00 (5.00–5.00)		-1.941	0.092	0.24
	Q3	4.22 (0.61)	4.00 (4.00–5.00)		4.41 (0.50)	4.00 (4.00–5.00)		-1.604	0.180	0.20
	Q4	4.16 (0.68)	4.00 (4.00–5.00)		4.38 (0.55)	4.00 (4.00–5.00)		-1.941	0.092	0.24
Knowledge and information sources	Q5	3.56 (0.80)	4.00 (3.00–4.00)	3.72 (0.47); 3.86 (3.36–4.11)	4.03 (0.75)	4.00 (4.00–5.00)	4.06 (0.40); 4.00 (3.75–4.43)	-2.982	0.003	0.38
	Q6	3.22 (0.66)	3.00 (3.00–4.00)		4.03 (0.60)	4.00 (4.00–4.00)		-4.025	0.000	0.51
	Q7	3.75 (0.92)	4.00 (3.25–4.00)		4.19 (0.54)	4.00 (4.00–4.75)		-2.401	0.016	0.30
	Q8	3.94 (0.91)	4.00 (4.00–4.75)		4.35 (0.61)	4.00 (4.00–5.00)		-2.162	0.030	0.27
	Q9	4.72 (0.80)	5.00 (5.00–5.00)		4.28 (0.77)	4.00 (4.00–5.00)		-2.566	0.012	0.34
	Q10	3.31 (0.78)	3.50 (3.00–4.00)		3.91 (0.59)	4.00 (4.00–4.00)		-3.189	0.001	0.40
	Q11	3.63 (0.83)	4.00 (3.00–4.00)		3.66 (0.87)	4.00 (3.00–4.00)		-0.243	1.000	0.03
Perceptions of patients and stopping medicines	Q12	3.47 (0.76)	3.50 (3.00–4.00)	3.54 (0.61); 3.58 (3.00–4.00)	3.97 (0.59)	4.00 (4.00–4.00)	4.04 (0.51); 4.00 (3.67–4.33)	-3.258	0.001	0.41
	Q13	3.56 (0.76)	4.00 (3.00–4.00)		4.13 (0.66)	4.00 (4.00–5.00)		-3.499	< 0.001	0.44
	Q14	3.58 (0.72)	4.00 (3.00–4.00)		4.03 (0.59)	4.00 (4.00–4.00)		-2.977	0.003	0.38
Perception of colleagues and stopping medicines	Q15	3.81 (0.54)	4.00 (4.00–4.00)	3.59 (0.29); 3.67 (3.36–3.77)	4.13 (0.49)	4.00 (4.00–4.00)	3.90 (0.46); 3.89 (3.58–4.11)	-1.955	0.063	0.24
	Q16	3.84 (0.45)	4.00 (4.00–4.00)		4.06 (0.72)	4.00 (4.00–4.75)		-1.334	0.241	0.17
	Q17	3.88 (0.61)	4.00 (3.25–4.00)		4.19 (0.54)	4.00 (4.00–4.75)		-2.106	0.051	0.26
	Q18	4.00 (0.37)	4.00 (4.00–4.00)		4.38 (0.61)	4.00 (4.00–5.00)		-2.668	0.011	0.34
	Q19	3.44 (0.62)	3.50 (3.00–4.00)		3.78 (0.61)	4.00 (3.00–4.00)		-2.294	0.034	0.29
	Q20	3.91 (0.39)	4.00 (4.00–4.00)		4.16 (0.69)	4.00 (4.00–5.00)		-1.789	0.116	0.23
	Q21	3.56 (0.56)	4.00 (3.00–4.00)		3.84 (0.72)	4.00 (3.00–4.00)		-1.8	0.108	0.23
	Q22	2.38 (0.94)	2.00 (2.00–3.00)		2.78 (1.34)	2.50 (2.00–4.00)		-1.796	0.070	0.22
	Q23	3.50 (0.92)	3.00 (3.00–4.00)		3.78 (0.91)	4.00 (3.00–4.00)		-1.416	0.180	0.18
Institutional factors	Q24	3.47 (0.84)	4.00 (3.00–4.00)	2.89 (0.60); 3.00 (2.40–3.40)	3.88 (0.79)	4.00 (3.00–4.75)	3.01 (0.79); 2.90 (2.20–3.70)	-2.166	0.038	0.27
	Q25	2.28 (1.11)	2.00 (1.00–3.00)		2.47 (1.44)	2.00 (1.00–4.00)		-1.292	0.227	0.16
	Q26	2.19 (1.12)	2.00 (1.00–3.00)		1.78 (0.87)	2.00 (1.00–2.00)		-2.207	0.035	0.28
	Q27	3.03 (0.82)	3.00 (2.25–4.00)		2.91 (1.33)	3.00 (2.00–4.00)		-0.608	0.552	0.08
	Q28	3.47 (0.62)	4.00 (3.00–4.00)		4.00 (0.80)	4.00 (4.00–4.75)		-3.532	< 0.001	0.44

^*^The probability *p* < 0.05 indicates that the difference is statistically significant

**Table 3:** Pre and post descriptive statistics of frequency by question and domain (data are n (%))

Domain	Questions	Strongly disagree		Disagree		Neither agree nor disagree		Agree		Strongly agree	
		pre	post	pre	post	pre	post	pre	post	pre	post
Perceptions around stopping medicines	Q1	0 (0%)	0 (0%)	4 (12.5%)	0 (0%)	8 (25.0%)	3 (9.4%)	16 (50%)	23 (71.9%)	4 (12.5%)	6 (18.8%)
	Q2	0 (0%)	0 (0%)	0 (0%)	0 (0%)	2 (6.3%)	1 (3.1%)	7 (21.9%)	2 (6.3%)	23 (71.9%)	29 (90.6%)
	Q3	0 (0%)	0 (0%)	0 (0%)	0 (0%)	3 (9.4%)	0 (0%)	19 (59.4%)	19 (59.4%)	10 (31.3%)	13 (40.6%)
	Q4	0 (0%)	0 (0%)	1 (3.1%)	0 (0%)	2 (6.3%)	1 (3.1%)	20 (62.5%)	18 (56.3%)	9 (28.1%)	13 (40.6%)
Knowledge and information sources	Q5	0 (0%)	0 (0%)	4 (12.5%)	1 (3.2%)	8 (25.0%)	5 (16.1%)	18 (56.3%)	17 (54.8%)	2 (6.3%)	8 (25.8%)
	Q6	0 (0%)	0 (0%)	4 (12.5%)	0 (0%)	17 (53.1%)	5 (16.1%)	11 (34.4%)	20 (64.5%)	0 (0%)	6 (19.4%)
	Q7	0 (0%)	0 (0%)	5 (15.6%)	0 (0%)	3 (9.4%)	2 (6.3%)	19 (59.4%)	22 (68.8%)	5 (15.6%)	8 (25.0%)
	Q8	0 (0%)	0 (0%)	4 (12.5%)	0 (0%)	2 (6.3%)	2 (6.5%)	18 (56.3%)	16 (51.6%)	8 (25.0%)	13 (41.9%)
	Q9	1 (3.4%)	0 (0%)	0 (0%)	1 (3.1%)	0 (0%)	3 (9.4%)	4 (13.8%)	14 (43.8%)	24 (82.8%)	14 (43.8%)
	Q10	0 (0%)	0 (0%)	6 (18.8%)	0 (0%)	10 (31.3%)	7 (21.9%)	16 (50%)	21 (65.6%)	0 (0%)	4 (12.5%)
	Q11	0 (0%)	0 (0%)	5 (15.6%)	4 (12.5%)	4 (12.5%)	7 (21.9%)	21 (65.6%)	17 (53.1%)	2 (6.3%)	4 (12.5%)
Perceptions of patients and stopping medicines	Q12	0 (0%)	0 (0%)	3 (9.4%)	0 (0%)	13 (40.6%)	6 (18.8%)	14 (43.8%)	21 (65.6%)	2 (6.3%)	5 (15.6%)
	Q13	0 (0%)	0 (0%)	3 (9.4%)	0 (0%)	10 (31.3%)	5 (15.6%)	17 (53.1%)	18 (56.3%)	2 (6.3%)	9 (28.1%)
	Q14	0 (0%)	0 (0%)	3 (9.7%)	0 (0%)	8 (25.8%)	5 (15.6%)	19 (61.3%)	21 (65.6%)	1 (3.2%)	6 (18.8%)
Perception of colleagues and stopping medicines	Q15	0 (0%)	0 (0%)	1 (3.1%)	0 (0%)	5 (15.6%)	2 (6.3%)	25 (78.1%)	24 (75.0%)	1 (3.1%)	6 (18.8%)
	Q16	0 (0%)	0 (0%)	0 (0%)	1 (3.1%)	6 (18.8%)	4 (12.5%)	25 (78.1%)	19 (59.4%)	1 (3.1%)	8 (25.0%)
	Q17	0 (0%)	0 (0%)	0 (0%)	0 (0%)	8 (25.0%)	2 (6.3%)	20 (62.5%)	22 (68.8%)	4 (12.5%)	8 (25.0%)
	Q18	0 (0%)	0 (0%)	0 (0%)	0 (0%)	2 (6.7%)	2 (6.3%)	26 (86.7%)	16 (50%)	2 (6.7%)	14 (43.8%)
	Q19	0 (0%)	0 (0%)	2 (6.3%)	0 (0%)	14 (43.8%)	10 (31.3%)	16 (50%)	19 (59.4%)	0 (0%)	3 (9.4%)
	Q20	0 (0%)	0 (0%)	0 (0%)	0 (0%)	4 (12.5%)	5 (16.1%)	27 (84.4%)	16 (51.6%)	1 (3.1%)	10 (32.3%)
	Q21	0 (0%)	0 (0%)	1 (3.1%)	1 (3.1%)	12 (37.5%)	8 (25.0%)	19 (59.4%)	18 (56.3%)	0 (0%)	5 (15.6%)
	Q22	6 (18.8%)	6 (18.8%)	12 (37.5%)	10 (31.3%)	10 (31.3%)	5 (15.6%)	4 (12.5%)	7 (21.9%)	0 (0%)	4 (12.5%)
	Q23	0 (0%)	1 (3.1%)	4 (12.5%)	1 (3.1%)	13 (40.6%)	8 (25.0%)	10 (31.3%)	16 (50%)	5 (15.6%)	6 (18.8%)
Institutional factors	Q24	0 (0%)	0 (0%)	5 (15.6%)	0 (0%)	9 (28.1%)	12 (37.5%)	16 (50%)	12 (37.5%)	2 (6.3%)	8 (25.0%)
	Q25	9 (28.1%)	10 (31.3%)	11 (34.4%)	11 (34.4%)	7 (21.9%)	1 (3.1%)	4 (12.5%)	6 (18.8%)	1 (3.1%)	4 (12.5%)
	Q26	10 (31.3%)	14 (43.8%)	11 (34.4%)	13 (40.6%)	8 (25.0%)	3 (9.4%)	1 (3.1%)	2 (6.3%)	2 (6.3%)	0 (0%)
	Q27	1 (3.1%)	7 (21.9%)	7 (21.9%)	6 (18.8%)	14 (43.8%)	4 (12.5%)	10 (31.3%)	13 (40.6%)	0 (0%)	2 (6.3%)
	Q28	0 (0%)	0 (0%)	2 (6.3%)	2 (6.3%)	13 (40.6%)	4 (12.5%)	17 (53.1%)	18 (56.3%)	0 (0%)	8 (25.0%)

**Table 4:** Pre and post descriptive statistics of frequency by domain (data are n (%))

Domain	Strongly disagree		Disagree		Neither agree nor disagree		Agree		Strongly agree	
	pre	post	pre	post	pre	post	pre	post	pre	post
Perceptions around stopping medicines	0 (0%)	0 (0%)	5 (3.9%)	0 (0%)	15 (11.7%)	5 (3.9%)	62 (48.4%)	62 (48.4%)	46 (35.9%)	61 (47.7%)
Knowledge and information sources	1 (0.5%)	0 (0%)	28 (12.7%)	6 (2.7%)	44 (19.9%)	31 (14.0%)	107 (48.4%)	127 (57.5%)	41 (18.6%)	57 (25.8%)
Perceptions of patients and stopping medicines	0 (0%)	0 (0%)	9 (9.5%)	0 (0%)	31 (32.6%)	16 (16.7%)	50 (52.6%)	60 (62.5%)	5 (5.3%)	20 (20.8%)
Perception of colleagues and stopping medicines	6 (2.1%)	7 (2.4%)	20 (7.0%)	13 (4.5%)	74 (25.9%)	46 (16.0%)	172 (60.1%)	157 (54.7%)	14 (4.9%)	64 (22.3%)
Institutional factors	20 (12.5%)	31 (19.4%)	36 (22.5%)	32 (20%)	51 (31.9%)	24 (15.0%)	48 (30%)	51 (31.9%)	5 (3.1%)	22 (13.8%)

### Perceptions around stopping medicines (D1)

There was a statistically significant pre-post improvement in participants’ confidence about stopping medicines safely for patients with a medium effect size (Z = -2.696, *p* = 0.008, *r* = 0.34) [Q1].

However, participants’ perceptions on whether stopping medicines can have a positive impact on patients’ health and wellbeing [Q2], on being confident in undertaking medication reviews [Q3] and on being confident in undertaking medication reviews with patients [Q4] did not significantly change as indicated by *p* values > 0.05. In addition, low effect sizes were recorded.

### Knowledge and information sources (D2)

There were statistically significant pre-post improvements in relation to knowledge and information sources for questions Q5-Q8 and Q10, with a high effect size for Q6 and medium effect sizes for Q5, Q7, Q9 and Q10 but a small effect size for Q8 and negligible effect size for Q11.

Participants were more aware of current evidence about stopping medicines (Z = -2.982, *p* = 0.003, *r* = 0.38) [Q5] and agreed to a greater extent that they had enough information to help make decisions about stopping medicines (Z = -4.025, *p* < 0.001, *r* = 0.51) [Q6].

Participants agreed to a greater extent that they would use e-learning to help make decisions about stopping medicines (Z = -2.401, *p* = 0.016, *r* = 0.3) [Q7], would use online information to help make decisions about stopping medicines (Z = -2.162, *p* = 0.03, *r* = 0.27) [Q8] and were more confident about deciding when it was appropriate to stop a medicine(s) (Z = -3.189, *p* = 0.001, *r* = 0.4) [Q10].

Participants’ willingness to increase their knowledge about stopping medicines safely was higher before the three ALS (pre-ALS median = 5, post ALS median = 4, Z = -2.566, *p* = 0.012, *r* = 0.34) [Q9].

On the other hand, participants’ agreement about fully understanding the medicines prescribed to their patients (including side effects) did not change [Q11].

### Perception of patients and stopping medicines (D3)

There were statistically significant pre-post improvements in relation to perception of patients and stopping medicines with medium effect sizes.

Participants were more confident about deciding when it was appropriate to stop medicines (Z = -3.258, *p* = 0.001, *r* = 0.41) [Q12], negotiating with patients about when to stop a medicine (Z = -3.499, *p* < 0.001, *r* = 0.44) [Q13] and on negotiating with patient’s family members (where appropriate) about when to stop medicines (Z = -2.977, *p* = 0.003, *r* = 0.38) [Q14].

### Perception of colleagues and stopping medicines (D4)

Non-significant pre-post differences with low effect sizes were recorded for the practitioners’ ability to negotiate decisions about stopping medicines with other practitioners/health care professionals where necessary (Z = -1.955, *p* = 0.063, *r* = 0.24) [Q15], their confidence in negotiating decisions about stopping medicines with colleagues in general (Z = -1.334, *p* = 0.241, *r* = 0.17) [Q16], with colleagues within their organisation (Z = -1.789, *p* = 0.116, *r* = 0.23) [Q20], with colleagues outside their organisation (Z = -1.8, *p* = 0.108, *r* = 0.23) [Q21] and with pharmacists (Z = -2.106, *p* = 0.051, *r* = 0.26) [Q17].

Likewise, the ALS tool did not significantly influence participants’ perceptions with respect to having enough time to negotiate decisions about stopping medicines with colleagues (Z = -1.796, *p* = 0.07, *r* = 0.22) [Q22] or their willingness to use e-learning to help in making decisions about stopping medicines with colleagues (Z = -1.416, *p* = 0.18, *r* = 0.18) [Q23].

However, there were significant positive pre-post improvements in confidence in negotiating decisions about stopping medicines with nurses (Z = -2.668, *p* = 0.011, *r* = 0.34) [Q18] and with hospital colleagues (Z = -2.294, *p* = 0.034, *r* = 0.29) [Q19] with medium and low (nearly medium) effect sizes, respectively.

### Institutional factors (D5)

Significant pre-post improvements with both low and medium effect sizes were found for the professionals’ feelings of being more supported making decisions about stopping medicines in their place of work (Z = -2.166, *p* = 0.038, *r* = 0.27) [Q24], their belief that other priorities will not get in the way of them being able to stop medicines (Z = -2.207, *p* = 0.035, *r* = 0.28) [Q26] and their confidence in making decisions about stopping medicines in different settings (e.g. care home, own home, hospital) (Z = -3.532, *p* < 0.001, *r* = 0.44) [Q28].

Conversely, non-significant changes with low and negligible effect sizes respectively, were recorded for having enough time to make decisions about stopping medicines (Z = -1.292, *p* = 0.227, *r* = 0.16) [Q25] and the availability of the necessary resources (e.g. equipment/staff) to support the process of stopping medicines for patients (Z = -0.608, *p* = 0.552, *r* = 0.08) [Q27].

### Post evaluation feedback survey

#### Qualitative analysis

The post evaluation feedback survey evaluated participants overall perceptions of the ALS, including their expectations, comprehension, what they found helpful/unnecessary, prospective application to practice and whether they would recommend it to colleagues.

**Fig. 1:**
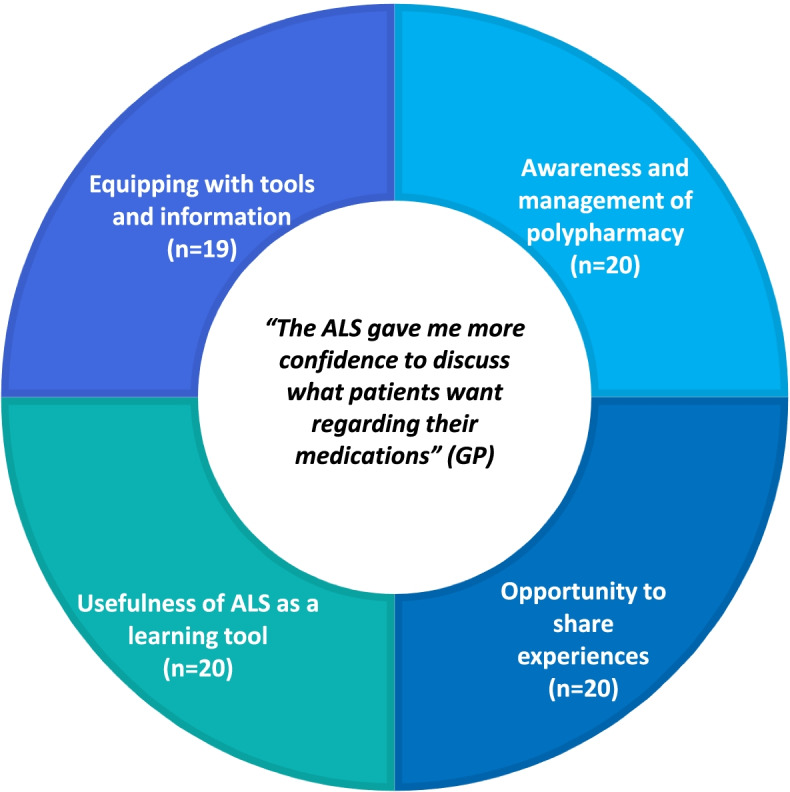
Top four themes with number of respondents who mentioned this theme in brackets

Analysis of qualitative responses from the survey revealed impact of the ALS tool across four key interrelated themes; i. awareness and management of polypharmacy; ii. opportunity to share experiences; iii. usefulness of ALS as a learning tool and iv. equipping with tools and information (Table 5) (Fig. 1). Themes are described in turn and illustrated through verbatim representative extracts from the survey. The extracts are labelled according to the type of participant: GP or pharmacy professional.

**Table 5:** Qualitative themes (QT) from post evaluation feedback survey questions

**Top four qualitative themes** **QT1- QT4**		**Post evaluation feedback survey question**	**Total number of items (*n* = 183)**	**Total number of respondents (*n* = 33)**
QT1	Awareness and management of polypharmacy	• Did the ALS meet your expectations? Please explain your answer • What would you most like to say about the ALS? • Were any parts of the ALS helpful? • How will you apply learning from the ALS to your practice? Give one important example	25 (14%)	20 (61%)
QT2	Opportunity to share experiences	• Did the ALS meet your expectations? Please explain your answer • What would you most like to say about the ALS? • Were any parts of the ALS helpful? • How will you apply learning from the ALS to your practice? Give one important example	36 (20%)	20 (61%)
QT3	Usefulness of ALS as a learning tool	• Did the ALS meet your expectations? Please explain your answer • What would you most like to say about the ALS? • Were any parts of the ALS helpful?	31 (17%)	20 (61%)
QT4	Equipping with tools and information	• Did the ALS meet your expectations? Please explain your answer • What would you most like to say about the ALS? • Were any parts of the ALS helpful? • How will you apply learning from the ALS to your practice? Give one important example	31 (17%)	19 (58%)

##### Awareness and management of polypharmacy (QT1)

Many participants reported how the ALS tool had increased their awareness and confidence in managing polypharmacy (for e.g. stopping inappropriate drugs), both in terms of their experiences over the duration of the ALS, but also prospectively, in relation to how they would apply learning from the ALS to their future practice.

*‘It has inspired me to be more proactive in the management of polypharmacy’ (GP)*

*“Looked at the different facets affecting the decisions surrounding polypharmacy e.g. when to stop, when to start and the personnel involved in these processes” (Pharmacy professional)*

*‘I will be more confident with reviewing and stopping medications for my elderly patients’ (GP)*

##### Opportunity to share experiences (QT2)

Participants stated how the ALS tool provided an opportunity to share experiences of managing polypharmacy with other attendees. This was perceived as valuable in gaining different professional perspectives and settings other than their own as well as providing time and a ‘safe’ space for reflection and discussion:

*‘Just having the time and space to discuss the issues especially in a multidisciplinary setting is useful’ (GP)*

*‘Good skills mix and interesting to see what problems different people may be facing when looking at the various aspects of deprescribing’ (Pharmacy professional)*

*Very useful. Non-judgemental and non-threatening environment. Good to share views (GP)*

Sharing learning from the ALS also featured as a future intention for some participants, in terms of disseminating learning with their colleagues from different professions and settings:

*‘Share information with GP colleague i.e. information for review/tools’ (Pharmacy professional)*

*‘Attend multidisciplinary team meetings/network with specialists in the areas I lack experience and confidence.’ (Pharmacy professional)*

##### Usefulness of ALS as a learning tool (QT3)

The usefulness of the polypharmacy ALS as a learning tool also featured as a core theme. Key elements included the style and presentation of the sessions:

*‘All three sessions were different; useful recapping at the beginning what had been covered. All the speakers were good and added to the action sets’ (GP)*

*‘It was very inspiring and well presented’ (GP)*

*‘Refreshing course, has helped me to learn an important and neglected area of general practice’ (GP)*

##### Equipping with tools and information (QT4)

Many respondents identified how the ALS tool equipped them with tools and information in managing polypharmacy both over the course of the ALS but also in terms of their future application of learning to practice:

*‘Has given me tools to help with deprescribing’ (GP)*

*‘They were very informative and provided insight into many aspects of the challenge of dealing with polypharmacy in patients’ (Pharmacy professional)*

*‘Gave me more insight, tools and confidence to discuss what patients want’ (GP)*

*‘Will use ‘Numbers needed to treat’ [concept] much more frequently’* (GP)

*‘Explore patient tools to visually explain the rationale behind stopping meds.’* (*Pharmacy professional)*

#### Quantitative analysis

In addition to the qualitative thematic findings, the majority of respondents (85%), reported that the polypharmacy ALS tool met their expectations, whilst 15% either reported that it had either not met or had not stated whether it had met their expectations. There was a high level of comprehension of the ALS; with 73% of respondents stating that there was not anything they did not understand during the ALS. Just over half or 55% of respondents stated that there were not any parts of the ALS which were unnecessary or not worthwhile, with the remaining respondents either not stating if there were any parts of the ALS they found unnecessary (24%) or (21%) indicating that they had found parts of the ALS unnecessary. These comments related to some parts being repetitive or suggestions to condense some sessions. Most respondents (97%) reported that they would recommend the polypharmacy ALS tool to colleagues.

Synthesised themes (ST) from analysis of the pre-post survey and the top four qualitative themes (QT) in the post evaluation feedback survey are provided in Table 6.

**Table 6:** Synthesised core themes (ST1-3) from pre-post ALS survey and post evaluation feedback survey

	Statistically significant statements (*n* = 14)^a^		Top four qualitative themes QT1- QT4	Synthesised themes ST1- ST3
Tool	Pre-post ALS survey		Post evaluation feedback survey	Pre-post ALS survey and post evaluation feedback survey
	Domains D1- D5	Statistically Significant Statements		
	Perceptions around stopping medicines (D1)	Q1. I am confident about stopping medicines safely for patients	Awareness and management of polypharmacy (QT1)	Awareness, confidence, and management of inappropriate polypharmacy (STI)
	Knowledge and information sources (D2)	Q5. I am aware of current evidence about stopping medicines Q6. I have enough information to help make decisions about stopping medicines Q7. I would use e-learning to help make decisions about stopping medicines Q8. I would use online information to help make decisions about stopping medicines Q10. I am confident about deciding when it is appropriate to stop a medicine(s)	Usefulness of ALS as a learning tool (QT3) Equipping with tools and information (QT4)	Equipping with knowledge, information, tools, and resources (ST2)
	Perception of patients and stopping medicines (D3)	Q12. I am confident about deciding when it is appropriate to stop medicines Q13. I am confident negotiating with patients about when to stop a medicine Q14. I am confident negotiating with patients’ family members (where appropriate) about when to stop medicines		
	Perception of colleagues and stopping medicines (D4)	Q18. I am confident negotiating decisions about stopping medicines with nurses Q19. I am confident negotiating decisions about stopping medicines with hospital colleagues	Opportunity to share experiences (QT2)	Decision-making and discussion about stopping medicines with colleagues in different settings (ST3)
	Institutional factors (Nixon and Vendelo, 2016) (D5)	Q24. I feel supported making decisions about stopping medicines where I work Q26. Other priorities do not get in the way of me being able to stop medicines Q28. I am confident about making decisions about stopping medicines in different settings (e.g. care home, own home, hospital)		

^a^Wilcoxon signed-rank test, *p* < 0.05 indicates that the difference is statistically significant

## Discussion

Building on the limited research and evaluation surrounding the role of initiatives in addressing inappropriate polypharmacy [8, 17], this study has demonstrated the impact of an educational polypharmacy ALS tool in improving healthcare practitioners’ awareness and confidence in this area. Analysis of pre-post survey data showed significant improvement in 14 statements, across all five domains including perceptions around stopping medicines (D1); knowledge and information sources (D2), perception of patients and stopping medicines (D3), perception of colleagues and stopping medicines (D4) and institutional factors (D5).

Analytical synthesis of findings from the pre-post ALS survey and post evaluation feedback survey, revealed the effectiveness of the ALS tool in accordance to three synthesised themes; Awareness, confidence and management of inappropriate polypharmacy (ST1); Equipping with knowledge, information, tools and resources (ST2) and Decision-making and discussion about stopping medicines with colleagues in different settings (ST3).

### Awareness, confidence, and management of inappropriate polypharmacy (ST1)

Due to the substantial medical, social and economic issues associated with inappropriate polypharmacy [1, 2, 5–10], the pre-post survey demonstrates the contribution of the ALS tool, in improving healthcare practitioners’ confidence around stopping medicines. The theme of awareness and management of polypharmacy identified through analysis of the post evaluation feedback survey substantiates this finding, with many participants reporting how the ALS tool increased their awareness and confidence in managing polypharmacy, over the duration of the ALS, but also prospectively in application of learning to practice in the future. However, though increased confidence and awareness in managing polypharmacy was identified more generally, in the pre-post survey, more specific areas including undertaking medication reviews and undertaking medication reviews with patients did not feature as areas of significant change. Neither did their perceptions on whether stopping medicines can have a positive impact on patients health and wellbeing.

### Equipping with knowledge, information, tools, and resources (ST2)

Pre-post survey results indicate significant improvements in relation to participants: being aware of current evidence; having enough information to help make decisions; use of e-learning and online information; desire to increase knowledge about stopping medicines and confidence about deciding when it is appropriate to stop medicines. The fourth theme of equipping with tools and information from analysis of post evaluation feedback survey findings, supports these findings, demonstrating the value of the ALS in providing knowledge through information, tools and resources to manage polypharmacy over the course of the ALS but also through future application of learning to practice. The third qualitative theme of usefulness of the ALS as a learning tool, also recognises the overall value of the ALS tool as a learning instrument, with many participants positively reporting upon its style and presentation. In addition, most participants reported that the ALS tool met their expectations, that it was comprehensive, and they would recommend it to colleagues.

### Decision-making and discussion about stopping medicines with colleagues in different settings (ST3)

Significant improvements in making decisions with nurses and hospital colleagues as well as about making decisions about stopping medicines in different settings (e.g. care home, own home, hospital) were evident in analysis of pre-post survey data. Though making decisions with colleagues more generally, or with pharmacists or colleagues within or outside their organisation did not constitute a significant area of improvement. The opportunity to share experiences with colleagues from other professional backgrounds and settings, featured as a qualitative theme in analysis of the post evaluation feedback survey, both in terms of participants perceptions and experiences during the ALS, but also in terms of future intentions in applying learning from the ALS, for example, through discussions with colleagues from other professions in multidisciplinary meetings. This improvement supports Mecca’s et al. [26] finding, through a mixed method study of an interprofessional and deprescribing intervention, that interprofessional experience was acknowledged to be of benefit to participants. Through demonstrating the value of the ALS to support decision making about stopping medicines for GPs and pharmacy professionals with other professional groups (e.g. nurses and hospital colleagues), these findings also offer the potential to raise awareness of the importance of deprescribing to all healthcare professionals, as well as families and caregivers involved in decision making [32].

In addition to ST1-ST3, other significant areas of improvement identified in analysis of the pre-post survey related to the domain of perception of patients and stopping medicines [D3], including confidence; in deciding when it is appropriate to stop medicines, negotiating with patients and negotiating with patients’ family members (where appropriate) about when to stop a medicine. These findings demonstrate the effectiveness of the ALS for respondents in supporting the patient-centred approach advocated by NICE recommendations [9, 15], the General Medical Council [16] and the Royal Pharmaceutical Society [17]. However, though significant improvements were identified in the analysis of all statements in the pre-post survey pertaining to D3, this did not feature as a key qualitative theme in the post evaluation feedback survey.

Pre-post survey analysis also indicated substantial improvements in participants feeling supported in making decisions about stopping medicines where they work as well as their perception of other priorities not getting in the way of them being able to stop medicines. Interestingly the concepts of support and other priorities, represent factors which are arguably extrinsic to an individual’s behaviour to influence, or contextual in nature [18], suggesting that the ALS may have positively influenced participants perception of their environment over the duration of attendance.

The results of this study must be interpreted with caution as they are subject to limitations arising from i. a small sample size, ii. participants’ limited occupation range, iii. the locality of the study, iv. unevidenced clinical benefit for patients, v. unevidenced longer term impact and vi. participants being an ‘engaged sample’. Due to the small size of the study and the greater representation of GPs in the sample compared to pharmacy professionals (75%/25%), further evaluation is required to examine the impact of the ALS upon the confidence, perceptions and experiences of healthcare practitioners from different professions within and across localities to enable further corroboration and synthesis of statistical and thematic findings. Limitations regarding sample size have been mitigated by reporting effect sizes. Moreover, further research is needed to evaluate the clinical benefit of the ALS tool for patients when applied in daily practice.

As the duration between the first and final ALS session was 1 month the study did not evaluate the longer-term impact of the polypharmacy ALS upon healthcare practitioners’ confidence, perceptions and experiences of stopping inappropriate medicines, which would provide insight into the extent to which evidence relating to the effectiveness of the ALS tool was applied and sustained.

It is recognised that by attending the ALS sessions, participating healthcare practitioners represent an ‘engaged sample’, who may already be aware and/or interested in improving their learning and practice of stopping inappropriate medicines.

## Conclusions

By demonstrating the effectiveness of the ALS tool in improving important areas such as healthcare practitioners’ awareness, confidence and perceptions in stopping inappropriate medicines, this paper contributes to developing an understanding of the role of educational initiatives in addressing inappropriate polypharmacy. Key areas of improvement include awareness, confidence and management of inappropriate polypharmacy, equipping with relevant knowledge, information, tools and resources and decision-making and discussion about stopping medicines with colleagues in different settings. In addition, the ALS tool was well received as an educational learning tool in terms of being comprehensive, meeting expectations and strongly recommended. The style and presentation of the ALS tool was also well received. Further research evaluating the impact of the ALS tool upon the perceptions and experiences of a variety of healthcare practitioners (e.g. nurse practitioners) as well as the longer-term impact are required to corroborate the findings.

## Data Availability

The data and materials are not available for open access, since their access is bound by the ethical agreement approved by the University of Southampton.

## Abbreviations

ALS: Action Learning Sets
D: Domain
HEE: Health Education England
IQR: Interquartile range
NIHR ARC: National Institute for Health Research Applied Research Collaborative
Q: Question
QT: Qualitative theme
SD: Standard deviation
ST: Synthesised theme
Wessex AHSN: Wessex Academic Health Science Network

## References

[1] Halli-Tierney AD, Scarbrough C, Carroll D (2019). Polypharmacy: evaluating risks and deprescribing. Am Fam Physician.

[2] Ong SM, Lim YMF, Sivasampu S, Khoo EM (2018). Variation of polypharmacy in older primary care attenders occurs at prescriber level. BMC Geriatr.

[3] Masnoon N, Shakib S, Kalisch-Ellett L, Caughey GE (2017). What is polypharmacy? A systematic review of definitions. BMC Geriatr.

[4] Ibrahim K, Cox NJ, Stevenson JM, Lim S, Fraser SDS, Roberts HC (2021). A systematic review of the evidence for deprescribing interventions among older people living with frailty. BMC Geriatr.

[5] Yorkshire and Humber AHSN Improvement Academy, Connected Health Cities. Effectiveness matters: reducing harm from polypharmacy in older people. Centre for Reviews and Dissemination, University of York; 2017. Available at: https://www.york.ac.uk/crd/publications/effectiveness-matters/polypharmacy/

[6] Helmy R, Zullig LL, Dunbar-Jacob J, Hughes DA, Vrijens B, Wilson IB, De Geest S (2017). ESPACOMP medication adherence reporting guidelines (EMERGE): a reactive-Delphi study protocol. BMJ Open.

[7] Payne RA, Abel GA, Avery AJ, Mercer SW, Roland MO (2014). Is polypharmacy always hazardous? A retrospective cohort analysis using linked electronic health records from primary and secondary care. Br J Clin Pharmacol.

[8] Duerden M, Avery T, Payne R. Polypharmacy and medicines optimisation: making it safe and sound. The Kings Fund; 2013. Available at: https://www.kingsfund.org.uk/publications/polypharmacy-and-medicines-optimisation

[9] National Institute for Health and Care Excellence (2009). Medicines adherence: Involving patients in decisions about prescribed medicines and supporting adherence (Clinical Guideline 76).

[10] World Health Organisation. The third WHO global patient safety challenge: medication without harm. Geneva; World Health Organisation: 2017. Licence: CC BY-NC-SA 3.0 IGO. Available at: www.gmc-uk.org/gmp

[11] Reeve E, To J, Hendrix I, Shakib S, Roberts MS, Wiese MD (2013). Patient barriers to and enablers of deprescribing: a systematic review. Drug Aging.

[12] Bokhof B, Junius-Walker U (2016). Reducing polypharmacy from the perspectives of general practitioners and older patients: a synthesis of qualitative studies. Drug Aging.

[13] Scott IA, Hilmer SN, Reeve E, Potter K, Le Couteur D, Rigby D, Gnjidic D, Del Mar CB, Roughead EE, Page A (2015). Reducing inappropriate polypharmacy: the process of deprescribing. JAMA Intern Med.

[14] Anderson K, Stowasser D, Freeman C, Scott I (2014). Prescriber barriers and enablers to minimising potentially inappropriate medications in adults: a systematic review and thematic synthesis. BMJ Open.

[15] National Institute for Health and Care Excellence (2015). Medicines optimisation: the safe and effective use of medicines to enable the best possible outcomes (Clinical Guideline NG5).

[16] General Medical Council. Good practice in prescribing and managing medicines and devices. General Medical Council; 2013. Available at: www.gmc-uk.org/gmp

[17] Royal Pharmaceutical Society (2019). Getting our medicines right.

[18] Duncan P, Cabral C, McCahon D, Guthrie B, Ridd MJ (2019). Efficiency versus thoroughness in medication review: a qualitative interview study in UK primary care. Br J Gen Pract.

[19] Rankin A, Cadogan CA, Patterson SM, Kerse N, Cardwell CR, Bradley MC, Ryan C, Hughes C (2018). Interventions to improve the appropriate use of polypharmacy for older people. Cochrane Database Syst Rev.

[20] Khalil H, Bell B, Chambers H, Sheikh A, Avery AJ (2017). Professional, structural and organisational interventions in primary care for reducing medication errors. Cochrane Database Syst Rev.

[21] Hilmer SN, Gnjidic D (2018). Deprescribing: the emerging evidence for and the practice of the ‘geriatrician’s salute’. Age Ageing.

[22] Ulley J, Harrop D, Ali, A, Alton, S, Fowler-Davis S. Deprescribing interventions and their impact on medication adherence in community-dwelling older adults with polypharmacy: a systematic review. BMC Geriatrics. 2019;19(1):15. Available at: 10.1186/s12877-019-1031-4 10.1186/s12877-019-1031-4 PMC6339421 30658576

[23] NHS Scotland (2020). The 7-Steps medication review.

[24] Pitkala KH, Juola AL, Kautiainen H, Soini H, Finne-Soveri UH, Bell JS, Bjorkman M (2014). Education to reduce potentially harmful medication use among residents of assisted living facilities: a randomized controlled trial. J Am Med Dir Assoc.

[25] Bregnhoj L, Thirstrup S, Kristensen MB, Bjerrum L, Sonne J (2009). Combined intervention programme reduces inappropriate prescribing in elderly patients exposed to polypharmacy in primary care. Eur J Clin Pharmacol.

[26] Mecca MC, Thomas JM, Niehoff KM, Hyson A, Jeffery SM, Sellinger J, Mecca AP, Van Ness PH, Fried TR, Brienza R (2019). Assessing an interprofessional polypharmacy and deprescribing educational intervention for primary care post-graduate trainees: a quantitative and qualitative evaluation. J Gen Intern Med.

[27] Nixon MS, Vendelo MT (2016). General practitioners’ decisions about discontinuation of medication: an explorative study. J Health Organ Manag.

[28] Cohen J (1988). Statistical power analysis for the behavioural sciences.

[29] Tomczak M, Tomczak E (2014). The need to report effect size estimates revisited. An overview of some recommended measures of effect size. Trends Sport Sci.

[30] Fritz CO, Morris PE, Richler JJ (2012). Effect size estimates: current use, calculations, and interpretation (vol 141, pg 2, 2011). J Exp Psychol Gen.

[31] Glaser B, Strauss A (1967). The discovery of grounded theory: strategies for qualitative research.

[32] World Health Organisation. Medication safety in polypharmacy. Technical report. Geneva: World Health Organisation; 2019. (WHO/UHC/SDS/2019.11). Licence: CC BY-NC-SA 3.0 IGO

